# An original and effective technique to improve exposure in open surgery

**DOI:** 10.1186/s10397-017-1013-5

**Published:** 2017-06-20

**Authors:** E. Nohuz, G. Chêne

**Affiliations:** 1 Department of Obstetrics and Gynecology, General hospital of Thiers, Route du Fau, 63300 Thiers, France; 2 EA 4681, PEPRADE, Université d’Auvergne, CHU Estaing, 1, place Lucie Aubrac, 63001 Clermont-Ferrand, France; 3 Department of Obstetrics and Gynecology, hôpital Femme-Mère-Enfant, HFME, Hospices civils de Lyon, CHU de Lyon, 59, boulevard Pinel, 69000 Lyon, France; 4 EMR 3738, Université Claude Bernard Lyon 1, 69000 Lyon, France

**Keywords:** Laparotomy, Myomectomy, Hysterectomy, Obstetric vacuum cup, Surgical technique

## Abstract

**Background:**

Exposure, especially when the organs are enlarged, remains one of the most important issue in open surgery. Considering this constraint appears critical in the progress of the surgical procedure. We highlight our technique which affords optimal exposure and improves manipulation and extraction of enlarged organs.

**Results:**

This original and effective technique is derived from an obstetrical device used to perform an assisted vaginal delivery. It improves exposure, reduces tissue manipulation, and enhances removal of the surgical specimen during hysterectomies and myomectomies. It can be similarly helpful sometimes to grasp and remove (by mini laparotomy) enlarged adnexa during laparoscopic procedures. Moreover, this trick appears particularly suited in case of obese patients.

**Conclusion:**

This new technique procures a real benefit for both the patient and the surgeon in terms of ergonomics and safety.

**Electronic supplementary material:**

The online version of this article (doi:10.1186/s10397-017-1013-5) contains supplementary material, which is available to authorized users.

## Backgound

Exposure, mainly when the organs are enlarged, remains one of the most important issue in open surgery. Taking into account, this constraint appears critical in the progress of the surgical procedure, in terms of ergonomics and security, particularly.

## Material and methods

In our practice, we use a trick with which exposure and maneuvering are facilitated. The use of a disposable Kiwi OmniCup® delivery device (Clinical Innovations®, Murray, Utah, USA) is a simple method to enhance exposure in open surgery, providing optimization of the operating space with adequate manipulation of the surgical specimen. This device, commonly used by obstetricians to perform an assisted vaginal delivery (instrumental extraction), is also used during a difficult fetal extraction by cesarean section [1–3]. It is a plastic, cup-shaped instrument which is applied to the fetal head after verification of the absence of vaginal wall (in case of vaginal delivery) and uterine wall (in case of cesarean section) in the suction area. As opposed to other surgical instruments, it does not increase the fetal head diameter for delivery. These advantages can be used in open surgery procedures. Indeed, the limited size of the device and the mobility of its traction system can release the operative field and facilitate the utero-adnexal exposure and extraction during open surgical procedures. Moreover, the laparotomy incision size is reduced because it is not necessary for the hands of the surgeon (or any instrument) to grasp the organ by surrounding it. Thus, this technique avoids the need for potentially hemorrhagic gests as traction sutures, or traumatic maneuvers, as surgical retractors whose use can lead to significant postoperative pain and even bowel, bladder, or parietal injuries. The use of a screw when performing a hysterectomy by laparotomy seems less ergonomic since this instrument does not allow all the degrees of freedom that the vacuum cup provides. In addition, the vacuum cup avoids any hysterotomy, and thus any tearing of the serous and the muscularis of the uterus. These points can thus represent advantages in terms of bleeding and carcinological safety. Additionally, the use of a single-use suction cup simplifies the sterilization logistics.

## Results and discussion

### Surgical technique (Additional file 1)

**Fig. 1:**
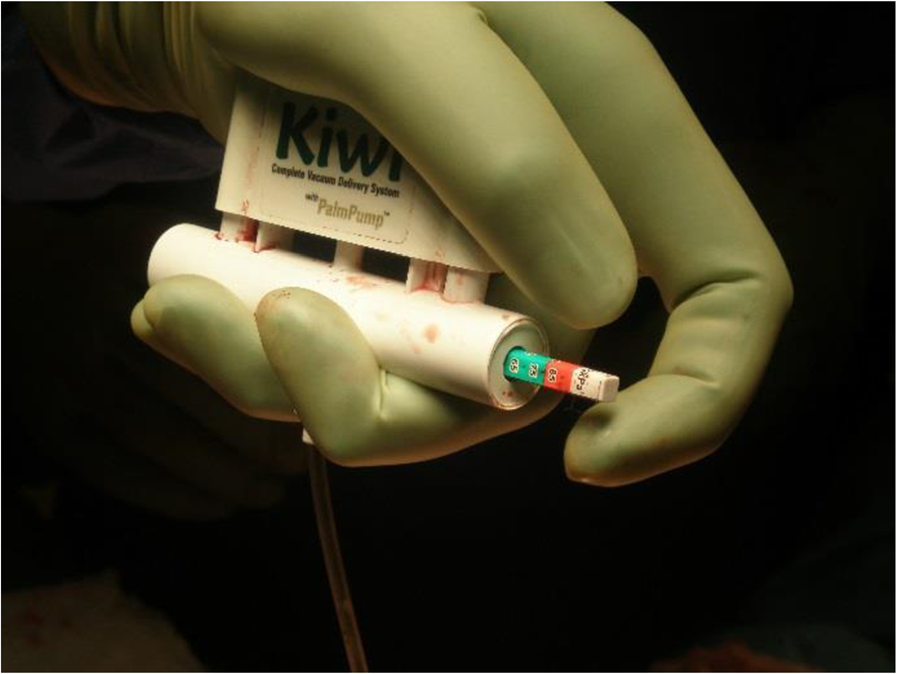
Obstetric device used during hysterectomy

**Fig. 2:**
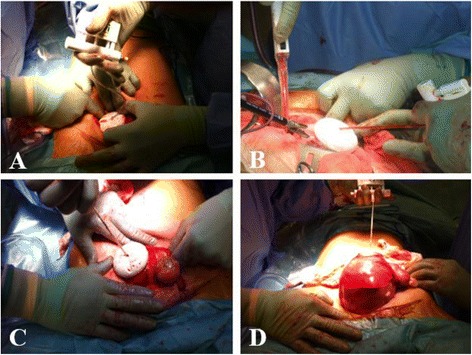
**a**–**d** Use of the vacuum cup during a laparotomic hysterectomy. The vacuum cup is positioned on the fundus of the uterus to tract it and permit exposition and dissection **a** and **b**. Divergent traction movements help to progressive extraction of the uterus **c** and **d**

We use a device of 50-mm diameter which provides the exposure of voluminous uterus during hysterectomies performed by laparotomy (Fig. 1). This size is commonly used in our obstetric practice. The vacuum cup is positioned on the fundus of the uterus to tract it, through the parietal incision. Its flexible-stem and low profile cup enable placement over the uterus no matter the depth of the operating space, making this technique particularly suited in an obese patient in whom the pelvic cavity offers a limited accessibility. This one is used as a real lever that allows the gripping while improving the dissection. Care must be taken to check the absence of tissue between the organ and the device (bowel, omentum, or fringes of the fallopian tubes) when applying the suction cup. This verification is visual but can sometimes be manual when the deepness of the operative field imposes it (surgeon’s fingers must circumscribe the contours of the suction cup before the priming of the vacuum). It is then necessary to manually activate the hand-held pump (connected to the vacuum cup by a hose) which also serves as a traction handle (manometer associating tactile sensation and traction force). Depressions of 600 to 700 mm Hg are generally sufficient for exposure and extraction. Divergent and progressive traction movements are exerted in-line with the parietal incision axis to grasp and extract the uterus comparably to the gestures performed during an assisted delivery (Fig. 2). It is sometimes necessary, when the procedure is relatively long in time, to re-prime the vacuum to continue the suction effect. The application of the suction cup requires a minimal size of the uterus, of the order of a fetal head diameter or more, to allow and preserve the leak-tightness of the suction chamber. Once the procedure has been completed, it is sufficient to engage the vacuum release valve, which is generally activated by the thumb to allow the release of the surgical specimen. In our experience, we also use this trick to perform myomectomy by laparotomy, after the uterus has been move out with the device. This one is removed and the hysterotomy is performed. Then, the suction cup is placed over the myoma after it had been sufficiently liberated to be taken by the device. Occasionally, this technique is similarly helpful to assist during the steps of grasping and removal (by mini-laparotomy) of some enlarged adnexa during laparoscopic procedures when the specimen cannot be placed into an endoscopic retrieval bag because of its large size and if a puncture should be avoided in terms of carcinological safety. However, this does not exempt the rules of good surgical practice (protection of the surgical field to prevent the consequences of a possible rupture of the cyst). Intended for the fetal scalp, the suction cup causes little trauma to the target organ.

## Conclusion

This original and effective technique is exploited to improve exposure, reduce tissue manipulation, and enhance removal of the surgical specimen. In this sense, it procures ergonomics and safety representing a real benefit for both the patient and the surgeon.


Additional file 1: Vacuum cup in laparotomy. (MP4 194252 kb)

